# Electron-beam FLASH whole brain irradiation induced a unique changes of intestinal flora

**DOI:** 10.1186/s10020-024-01053-w

**Published:** 2025-05-02

**Authors:** Feifei Gao, Wei Cheng, Yanxi Ma, Boyi Yu, Xinle Lang, Xiaodong Jin, Jianxin Wang, Xianhong Liu, Cuixia Di, Hui Wang, Fei Ye, Ting Zhao, Weiqiang Chen, Qiang Li

**Affiliations:** 1 Institute of Modern Physics, Chinese Academy of Sciences, Lanzhou, 730000 China; 2 Key Laboratory of Heavy Ion Radiation Biology and Medicine of Chinese Academy of Sciences, Lanzhou, 730000 China; 3 Key Laboratory of Basic Research on Heavy Ion Radiation Application in Medicine, Lanzhou, 730000 Gansu Province China; 4 University of Chinese Academy of Sciences, Beijing, 100049 China; 5 Institute of Applied Electronics, China Academy of Engineering Physics, Mianyang, 621900 China; 6 Zhongjiu Flash Medical Technology Co., Ltd., Mianyang, 621000 China

**Keywords:** E-beam FLASH, FLASH effect, Intestinal flora, Transcriptomics, Whole-brain irradiation

## Abstract

**Background:**

Whole-brain radiotherapy (WBRT) is an important way to treat multiple metastases. Ultra-high dose rate (FLASH) can avoid neurotoxicity caused by conventional irradiation, it has attracted much attention. This study aims to study the difference of irradiation-induced intestinal flora between conventional dose rate and FLASH WBRT.

**Methods:**

WBRT with 10 Gy was performed with electron-beam conventional irradiation (2 Gy/s) and electron-beam FLASH (eFLASH) irradiation (230 Gy/s). The intestinal feces and whole brain of mice were isolated after behavioral evaluation at 1st, 3rd and 10th weeks post-irradiation. HE staining and immunofluorescence were used to access the level of brain damage. The differences in intestinal microbes and transcription levels were detected by 16S rRNA gene sequencing and transcriptome sequencing, respectively.

**Results:**

eFLASH irradiation significantly reduced radiation neurotoxicity and had a long-term protective effect on cognitive function and learning and memory ability. Compared with conventional irradiation, eFLASH irradiation not only up-regulated the expression of genes related to neuronal regeneration and digestive system, but also induced more abundant intestinal microflora, especially the “probiotics” such as *Lachnospiraceae* and others, which were proved to play a role in radiation protection, increased significantly after eFLASH irradiation. The up-regulated microbiota after eFLASH irradiation was significantly positively correlated with genes related to neuronal development and regeneration, while significantly negatively correlated with genes related to inhibitory synapses. Additionally, conventional irradiation down-regulated microbial metabolism-related pathways, while FLASH did not.

**Conclusions:**

In summary, we explored the unique gut microbiota changes induced by eFLASH WBRT for the first time, providing a theoretical basis for exploring the mechanism of action of FLASH.

**Supplementary Information:**

The online version contains supplementary material available at 10.1186/s10020-024-01053-w.

## Background

Whole brain radiotherapy (WBRT) is the key to the treatment of multiple brain metastases, especially the number of brain metastases exceeds three. However, studies (Li and Brown 2017; Mehta et al. 2017) have shown that WBRT leads to a decline in neurocognitive function in patients, especially affecting short-term and long-term memory, and reducing the quality of life. This decline is related to damage to intracranial hippocampal structure. Researchers are currently exploring methods to protect the hippocampus during whole-brain radiotherapy to mitigate neurocognitive decline.

Flash radiotherapy (FLASH-RT) is an innovative technique, which is defined by ultra-high dose rate (≥ 40 Gy/s) (Lin et al. 2022). Compared to conventional radiotherapy (COVN), FLASH-RT can significantly reduce toxicity to normal tissues while maintaining similar anti-tumor efficacy, known as the FLASH effect. Preclinical studies have confirmed that FLASH-RT can effectively reduce toxicity in lung, intestine, brain and skin, while preserving its anti-tumor effects (Simmons et al. 2019; Fouillade et al. 2020; Ruan et al. 2021; Shi et al. 2022; Favaudon et al. 2014). As these encouraging results, FLASH is now recognized as one of the most promising breakthroughs in the field of radiation oncology. However, so far, the biological mechanism of FLASH effect is very complex. At present, several hypotheses have been proposed to explain FLASH effect, such as oxygen depletion and immune hypothesis, but they are not perfect.

The gut is often referred as the “second brain” of the human body due to the bidirectional communication between the gut and brain, known as the “gut-brain axis”. Studies have shown that the brain can send signals to the intestine through neural pathways (such as the vagus nerve pathway, enteric nervous system), endocrine pathways (such as the thalamic–pituitary–adrenal axis, HPA, neuropeptides) and immune pathways (the regulation of the brain to the immune system, the feedback effect of the intestinal immune system) (Liu et al. 2021a; Ge et al. 2021). Gut-brain axis is closely related to various mental illnesses, with gut flora influencing brain function and mood through the production of neurotransmitters such as 5-hydroxytryptamine and short-chain fatty acids (SCFAs). Conversely, mental stress and emotional states can impact gut health (Chu et al. 2019; Bove and Travagli 2019; Rutsch et al. 2020).

Intestinal microbes, which serve as a bridge of communication with the host, are highly susceptible to environmental factors. Numerous studies have shown that radiation exposure can alter gut microbes (Liu et al. 2021b; Kim et al. 2015). Most research in this area has focused on changes in the gut microbiota from direct exposure to whole-body, pelvic or abdominal irradiation. For instance, a study by Mitra et al. (2020) found the diversity of intestinal flora decreased in patients undergoing pelvic radiotherapy as treatment progressed. Few studies have examined the effects of partial irradiation away from the intestine on the intestinal microflora. Wang et al. (2024) discovered changes in the structure of intestinal flora following intracranial irradiation in mice, including a reduction in *Akkermansia* which is negatively correlated with autism, and an increase in *Parabacteroides* found in the feces of patients with depression. They believe that the brain and the gut use serum as a bridge for signal transduction through metabolites. However, the study lacked behavioral validation and only speculated that intracranial irradiation might alter intestinal microbiota by affecting mouse behavior.

To date, the impact of FLASH WBRT irradiation on intestinal flora has not been reported. Therefore, in this study, we performed behavioral and gut microbiota analysis following a single 10 Gy irradiation of the whole brain of mice. To test whether the FLASH effect causes different changes in intestinal microbiota compared with CONV.

## Methods

### Mice and irradiation devices

Female C57BL/6 mice (aged 6–8 weeks old) were purchased from Lanzhou Veterinary Research Institute, Chinese Academy of Agricultural Sciences. All animal experiments followed the principles of animal ethics and were approved by the Ethics Committee for Animal Experiments of the Institute of Modern Physics, Chinese Academy of Sciences No. 2023 (006) and were conducted within the guidelines of the institution.

The radiation device used in this study is a 9 MeV electron accelerator developed by Institute of Applied Electronics, China Academy of Engineering Physics. The accelerator is drived by a magnetron and consists of a microwave thermionic electron gun and a S-band linear accelerator module. The beam is led out of the vacuum from a titanium window. The repetion frequency is adjustable from 1 to 250 Hz and the pulse width is 3.5 µs.

### Whole-brain irradiation

**Fig. 1:**
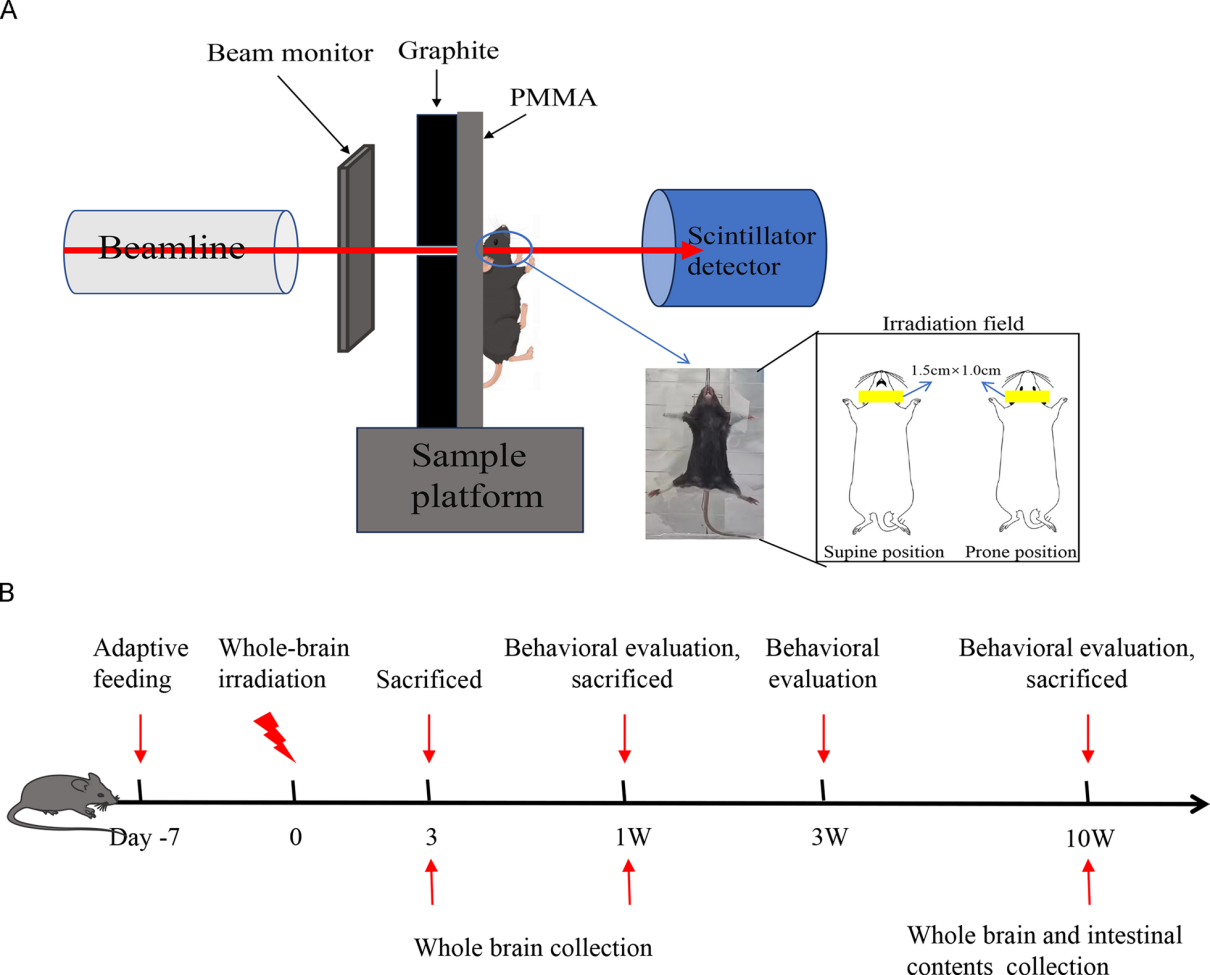
Whole-brain irradiation. **A** Irradiation device; **B** Flow chart of experiment

The mice were anesthetized (1% Avertin; 0.1 mg/20 g body weight) and fixed on a fixation plate and then the fixed mice were placed directly behind (in contact with) the graphite, so that the irradiation field irradiated the entire brain area at 1.5 cm × 1.0 cm, while limiting the dose to the eyes, mouth and other parts of the body (Fig. 1A). For all experiments, mice were divided into three groups (CK, CONV and FLASH) and received a single dose of 10 Gy (irradiation parameters in Table 1, n = 15–16/group).

**Table 1:** Irradiation parameters

	Dose (Gy)	Average dose rate (Gy/s)	Repeated frequency (Hz)	Number of pulses	Irradiation time (s)
CONV	10	≈ 2	2	11	5
FLASH	10	230	250	11	0.04

### Hematoxylin–eosin (HE) staining and immunohistochemistry

The whole brain tissue was taken out on the 3rd day after irradiation, fixed with 4% paraformaldehyde and then dehydrated, embedded, sliced, dewaxed, hydrated, stained, differentiated, dehydrated, transparent, sealed, dried naturally, finally placed under the microscope to observe the structural morphological changes and photographed. NeuN and Iba1 were used to access neuronal damage and the number of microglia, respectively. Floating brain sections were incubated with anti-NeuN (1:2000, GB11138, Servicebio) and anti-Iba1 (1:3000, GB113502, Servicebio) primary antibodies. HRP-labeled goat anti-rabbit IgG (1:500, GB23303, Servicebio secondary antibodies were used. IF555-Tyramide (1∶500, G1233) and iF488-Tyramide (1:500, G1231) were used for staining, respectively. NeuN and Iba1 expression area were quantified using ImageJ software.

### Behavioral tests

We conducted behavioral experiments in mice at 1st, 3rd, and 10th weeks after irradiation, respectively. All behavioral experiments were conducted on the software VisuTrack. The details as follows.

The open field test (OF) was used to assess voluntary movement, exploratory activity and general anxiety. Each mouse was placed in the OF arena (1 × 1 m) for 5 min and allowed to explore freely. The total distance traveled, immobility time and others were recorded.

Rotarod test: the mice were placed on a mouse rotating rod fatigue apparatus to gradually accelerate from 5 to 40 rpm, culminating in a 5-min endurance training session at 40 rpm, and the time at which the mice dropped the rotating rod was recorded.

Novel object recognition (NOR) evaluated the cognitive memory ability of mice by exploring the length of time between familiarize objects and new unfamiliar objects. Each mouse was allowed to familiar with two identical objects for 5 min in OF. 1 h later, mice were placed with a new object and the same familiar object for 5 min. The time and times spent exploring each object were measured and the recognition ratio (RR) was calculated. Formula: RR = (times spent exploring the new object)/(times spent exploring both objects) × 100.

Forced swimming test (FST) (Mitra et al. 2020) was used to assess the depression level of mice. Each animal was placed into a transparent cylinder (25 cm high, 12 cm in diameter), containing 15 cm of water at 25 ± 2 ℃. During a 6-min experiment, immobility and climbing time throughout the last 4 min was recorded.

Y maze training was used to test the learning memory behavior of mice. Spontaneous alternation was measured in a single 8 min trial in a standard Y-maze made of clear acrylic tubing, with arms 32 cm long. Alternation was defined as consecutive entries into three different arms. Formula: alternation rate% = number of successful alternations/(total number of entries − 2) × 100.

Morris water maze (MWM) is the most widely used behavioral experiment to evaluate the spatial learning and memory ability of animals. Two phases: place navigation test (PNT) and spatial probe test (SPT). We exposed the platform to the water on the first day and recorded the average speed to reach the platform, which was used to exclude its own disturbing factors. PNT: the platform was placed below the surface of the water and the mice were randomly placed in four locations: east, west, south and north. The time from placing the mice into the water to finding the underwater platform was recorded. If the time to find the underwater platform exceeded 120 s, the animal was guided to the platform and then was allowed to stay on the platform for 10 s. Each animal was trained 4 times a day, with an interval of 60 min between the two training sessions for 4 consecutive days. SPT: The platform was removed and the mouse was placed into the water from the opposite side of the original platform quadrant. The escape latency time (i.e., the time the animal first found the underwater platform after entering the water) and the number of platform crossings were recorded.

### mRNA sequencing

On the seventh day after irradiation, the whole brain of the mice was taken out and frozen in liquid nitrogen and stored at – 80 ℃. Transcriptome sequencing and analysis were performed by Shanghai OE Biotech Co., Ltd. (Shanghai, China). Differential expression analysis was performed using the R package DESeq2. Q-value < 0.05 and foldchange > 2 or foldchange < 0.5 was set as the threshold for significantly differential expression genes (DEGs). Based on the hypergeometric distribution, GO and KEGG (Kyoto Encyclopedia of Genes and Genomes, http://www.genome.jp/kegg/) enrichment analysis of DEGs were performed to screen the significant enriched term using R (v3.2.0), respectively.

### 16S rRNA sequencing and microbial diversity analysis

Samples of intestinal contents were rapidly frozen after collection and stored at – 80 ℃. The composition of the intestinal microbiota was analyzed based on the 16S rRNA sequences detected by Shanghai OE Biotech Co., Ltd. Based on ASVs (amplicon sequence variants), community diversity, species difference and functional prediction analysis were conducted. Alpha-diversity was based on Chao1, Shannon, ACE and Simpson index to analyze the complexity of species diversity of each microbial community. Beta-diversity analysis was performed using principal co-ordinates analysis (PCoA) based on Binary-Jaccard distance. For functional annotation of gut microbes, 16S rRNA gene sequencing data were compared with KEGG functional pathway databases. Wilcoxon test was used to test for species and phenotypic differences between the two groups and Kruskal–Wallis used for the three, the filtering threshold is p-value < 0.05. Linear discriminant analysis (LDA) coupled with effect size measurements (LEfSe), which reveals the composition of different species of two or more groups of biomes.

### Correlation analysis and statistics

Correlation analysis shows the correlation of features between different omics. Spearman correlation algorithm was used in this analysis and the screening criterion was p-value < 0.05. Statistical analyses were performed using GraphPad Prism version 8.0 and Excel(2016). Three groups were performed using ANOVA and Kruskal–Wallis. The two groups were compared using student’s t-test and Wilcoxon test. All data are presented as the mean ± standard error of the mean.

## Results

### FLASH irradiation reduces radiological acute injury

**Fig. 2:**
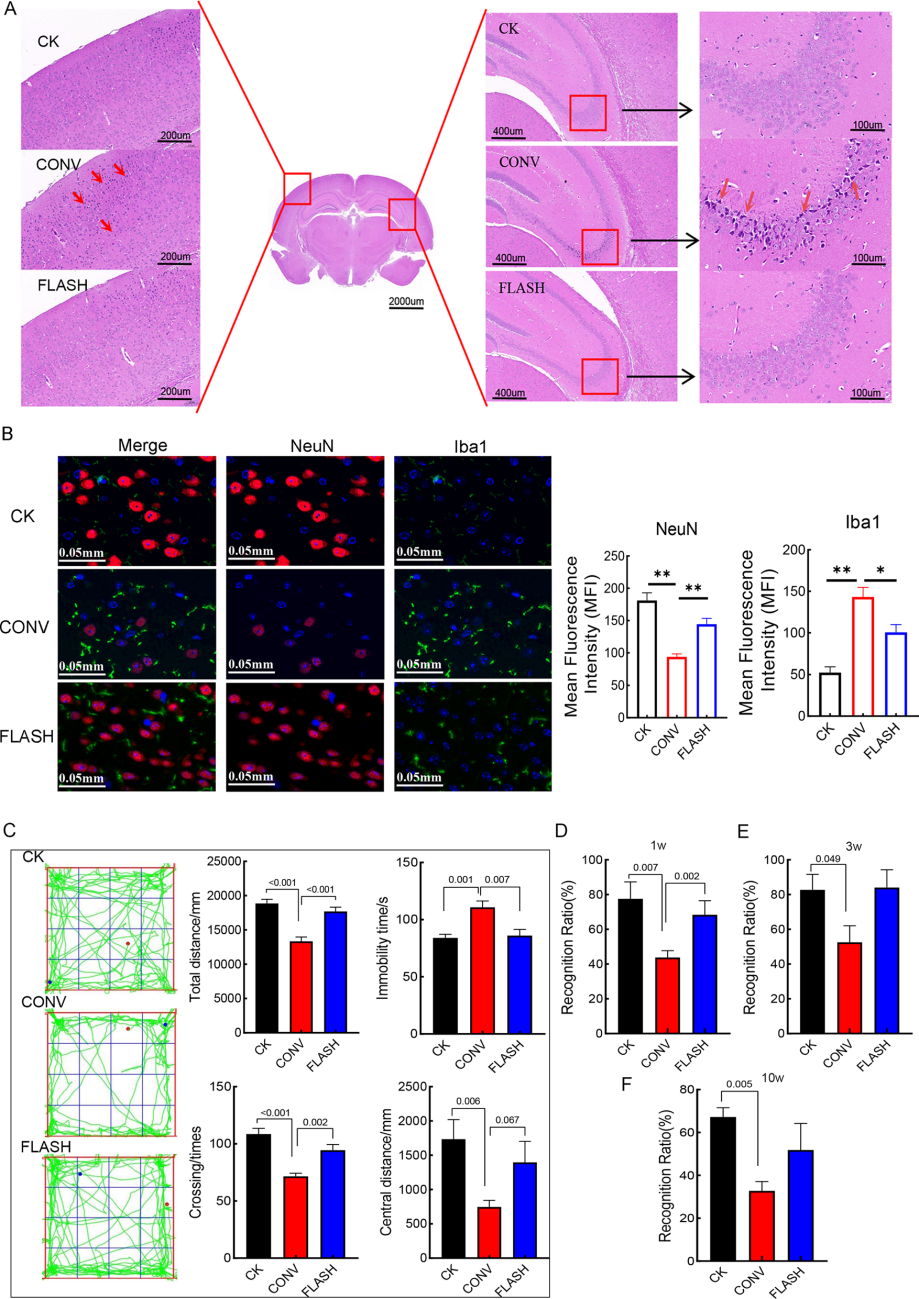
FLASH irradiation reduces radiological acute injury. **A** HE staining. **B** Immunofluorescence of NeuN and Iba1. **C** The OF test 1 week post-irradiated; D-F. The NOR test 1, 3, 10 week post-irradiated. The differences between the two were analyzed by student’s t- test

Figure 1B illustrates the whole experimental flow. The HE results revealed significant neuronal degeneration and reactive glial cell proliferation in the hippocampus, temporal lobe, frontal lobe and other regions of mice subjected to conventional irradiation (CONV), whereas these effects were markedly reduced in the FLASH irradiation group (Fig. 2A, Figure S1A, red arrow). Additionally, brain tissue exposed to CONV exhibited a loose and irregular arrangement (Figure S1A, yellow arrow). To determine the effect of each irradiation way on nerve damage, we quantified the number of microglia (Iba1) and neurons (NeuN). The results showed that the CK group showed the strongest NeuN fluorescence and the number of neurons was healthy, while the fluorescence in the CONV group almost disappeared, and the fluorescence in the FLASH group was weakened but stronger than that in the CONV group, indicating that the neuronal damage in the FLASH group was alleviated. In addition, compared with CK and FLASH groups, the fluorescence of Iba1 in CONV group was enhanced and the number was increased (Fig. 2B). This indicated that there was more gliosis in the CONV group, indicating more damage.

The 1st week behavior results showed that CONV caused more severe impairments in movement and cognitive functions in mice, whereas FLASH irradiation resulted in no significant changes. This was evidenced by an increase in total distance travelled, the number of crossing and the central distance, along with a decrease in immobility time (Fig. 2C), and an improvement in the recognition ratio (Fig. 2D) compared with CONV. Both pathological and behavioral assessments suggest that FLASH irradiation mitigates radiation-induced acute brain injury.

### Long-term protective effects of FLASH on cognition and learning memory

In the OF test at 3rd and 10th week post-irradiation, there were no significant changes in movement metrics among CK, CONV and FLASH group (Figure S1B, C). To verify this, we added rotarod test at 10th week and the results were consistent with the OF test (Figure S1D). The cognitive impairment caused by CONV was still significantly different from the CK and FLASH irradiated groups (Fig. 2E, F).

**Fig. 3:**
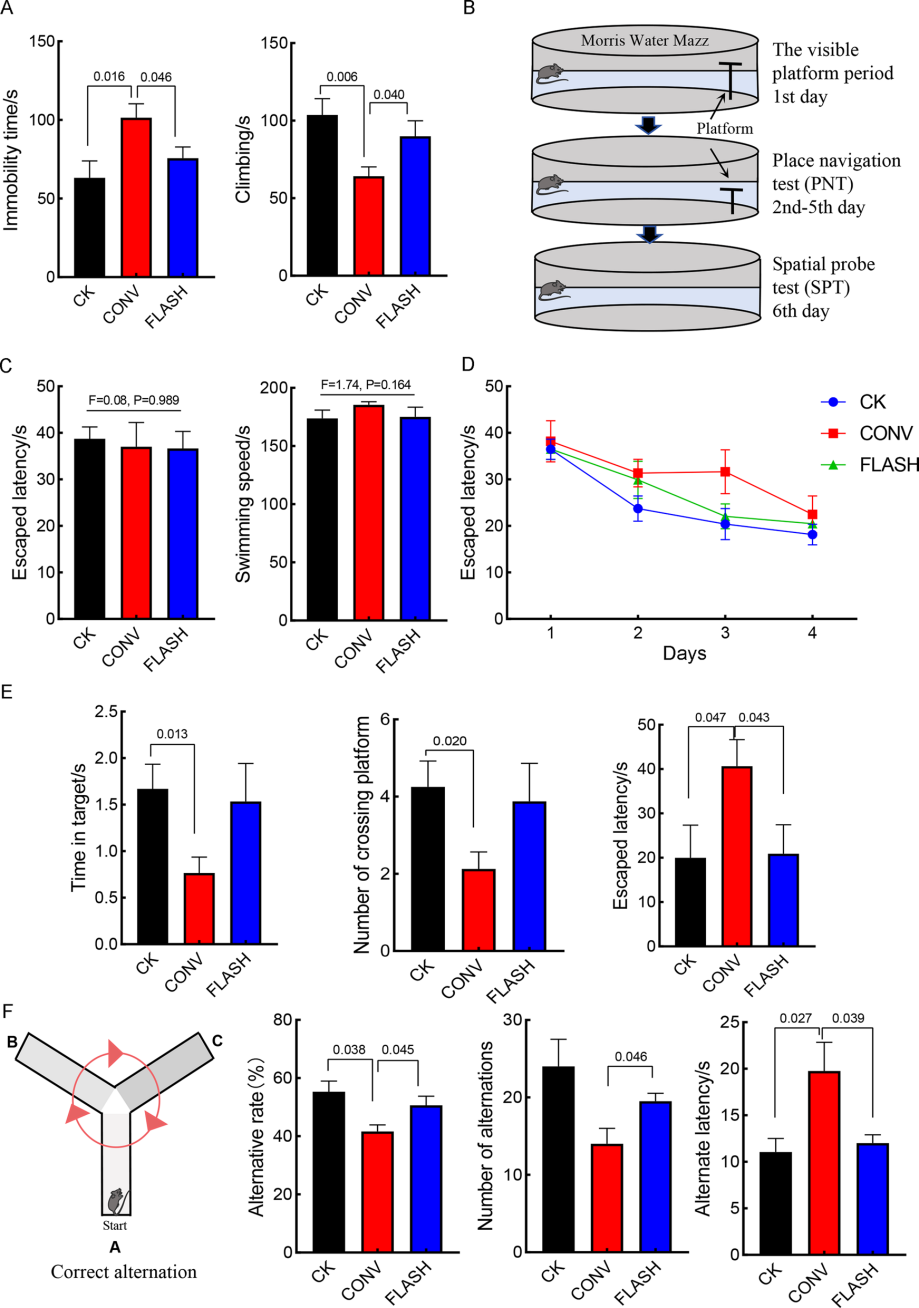
Long-term protective effects of FLASH. **A** FST in the 3rd week after irradiation; **B** Morris Water Maze in the 3rd week after irradiation; **C** Visible platform period of MWM; **D** PNT of MWM; **E** SPT of MWM; **F** Y maze in the 10th week after irradiation

Additionally, we incorporated FST and MWM at the 3rd week, and Y maze at the 10th week, to evaluate the long-term protective effect of FLASH on cognitive and learning memory functions in mice. The FST results showed that at the 3rd week, mice exposed to CONV irradiation had a significantly increased immobility time and reduced climbing time, indicating increase level of the depression, while FLASH irradiation did not (Fig. 3A).

The MWM results are shown in the Fig. 3B. During the visible platform phase, there was no significant difference in the latency and swimming speed among the mouse, ruling out individual differences (Fig. 3C). Subsequently, we conducted a 4-day PNT. On the third day, the latency reach the platform was longer in the CONV group compared to the CK and FLASH groups (Fig. 3D). The SPT results indicated that the CONV group had the longest latency to the platform, the shortest time spent near the platform, and least entries into the platform area. Thus, the spatial learning ability of mice in the CONV group was impaired, whereas there was no difference between the FLASH and CK groups (Fig. 3E).

At the 10th week, due to the poor condition of the mice, we used Y-maze to evaluate the spatial working memory. The results showed that the CONV had the lowest alternation rate and the longest alternation latency among the three arms, with no difference between the FLASH group and CK groups. This provides further evidence for the long-term protective effect of FLASH on spatial learning and memory function in mice (Fig. 3F).

### FLASH irradiation up-regulated the digestive system-related mRNA expression

**Fig. 4:**
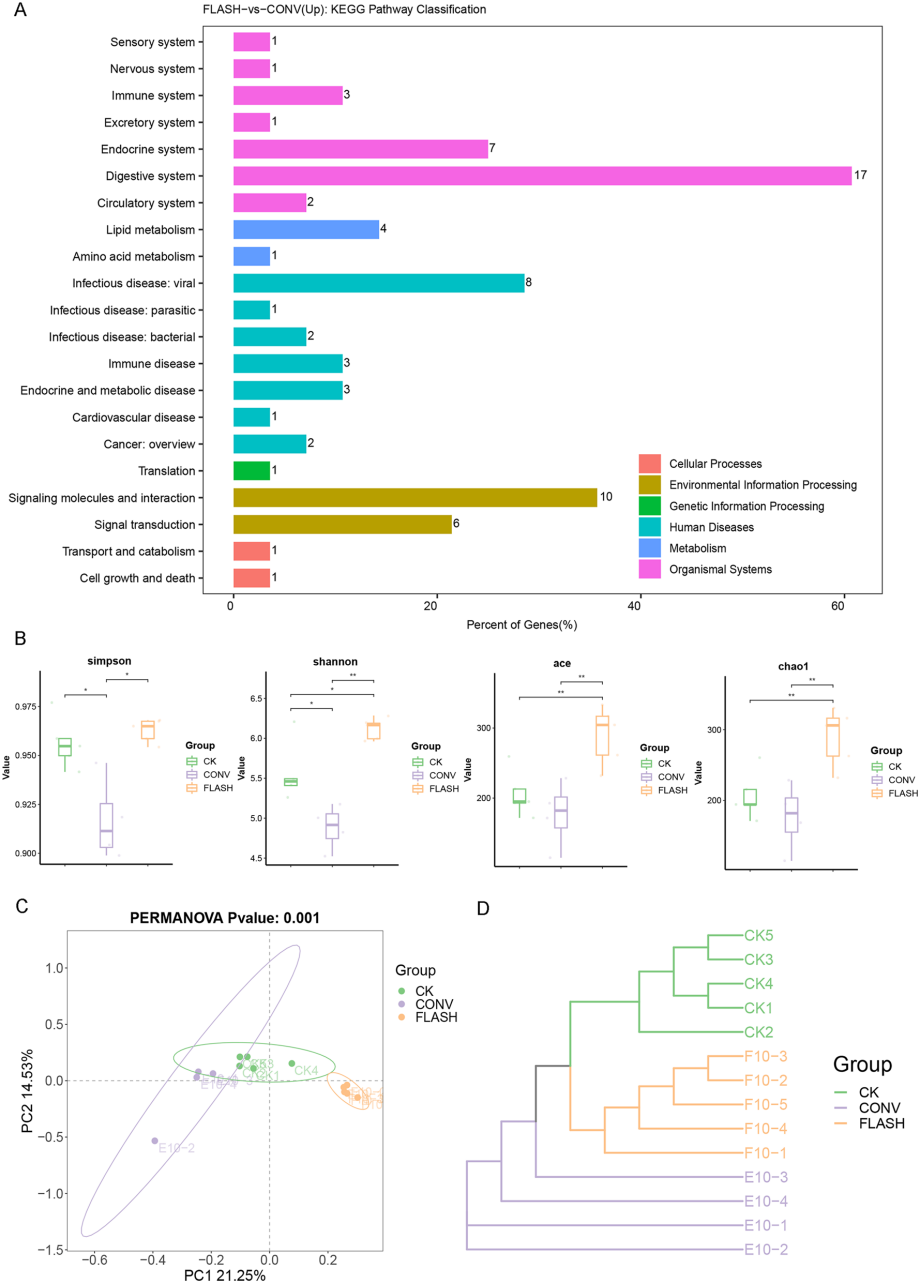
Transcriptome and gut microbiota. **A** Transcriptome KEGG enrichment analysis; **B** Microbial-diversity; **C**, **D** Beta-diversity based on PCoA maps and UPGMA hierarchical clustering tree. “*” means p < 0.05, “**” means p < 0.01, “***” means p < 0.001)

KEGG enrichment analysis revealed that the up-regulated genes in the FLASH group were primarily enriched in pathways related to the digestive system (Fig. 4A). These pathways include pancreatic secretion, fat, protein and vitamin digestion and absorption, as well as metabolic pathways such as tyrosine metabolism and glycerolipid metabolism (Figure S2A). GO enrichment analysis indicated that the up-regulated genes in the FLASH group were predominantly associated with processes such as lipid catabolic process and serine-type endopeptidase activity (Figure S2B). These pathways are related to microbial metabolism, suggesting that the FLASH effect may involve the participation of microorganisms. In addition, we compared the CONV group with FLASH and CK groups, respectively. GO enrichment results showed that CONV irradiation up-regulated the genes related to hydrogen peroxide catabolic process, negative regulation of appetite, oxygen carrier activity, peroxidase activity and others compared with CK (Figure S4A). However, genes about growth factor binding and serine peptidase activity were up-regulated in FLASH group compared with CK (Figure S4B). We then calculated the GSEA enrichment scores of related pathways between the different groups and found that the complement and coagulation cascades pathways were significantly up-regulated in the CONV group compared with CK (NES: 1.4636). However, the enrichment score in FLASH group was smaller and not statistically significant. For chemical carcinoma-DNA adducts (mmu05204), although there was no statistical difference between the two comparison groups, it was up-regulated in the CONV group, but down-regulated in the FLASH group (Figure S4C), indicating that the FLASH group produced less toxic substances.

### FLASH irradiation caused less alteration of the gut microbiota

The sparse curve of the analyzed samples demonstrated a high number of unique sequence variations, indicating substantial sample coverage and species richness. Good’s coverage for all three groups was close to 1 (Figure S3A, B). These results suggest that the sequencing data were sufficient to capture and reveal most microbial communities in all analyzed samples.

Microbial α-diversity was assessed using Simpson, Shannon, Chao1, Ace indices. After CONV irradiation, the α-diversity index was significantly lower than that of CK and FLASH groups, indicating that FLASH irradiation caused less alteration of the gut microbiota (Fig. 4B).

Regarding β-diversity (Fig. 4C, D), significant differences were observed between CONV and FLASH groups in the PCoA maps. To further analyze the similarity and differences in microbial species distribution among the samples, a weighted UPGMA hierarchical clustering tree was constructed, revealing significant differences among the three groups. Additionally, the Rank-Abundance curve showed a long tail distribution (Figure S3C), indicating that the intestinal flora of the analyzed samples was rich in ASVs and these ASVs were evenly distributed.

### FLASH exposure increased the abundance of “probiotics”

**Fig. 5:**
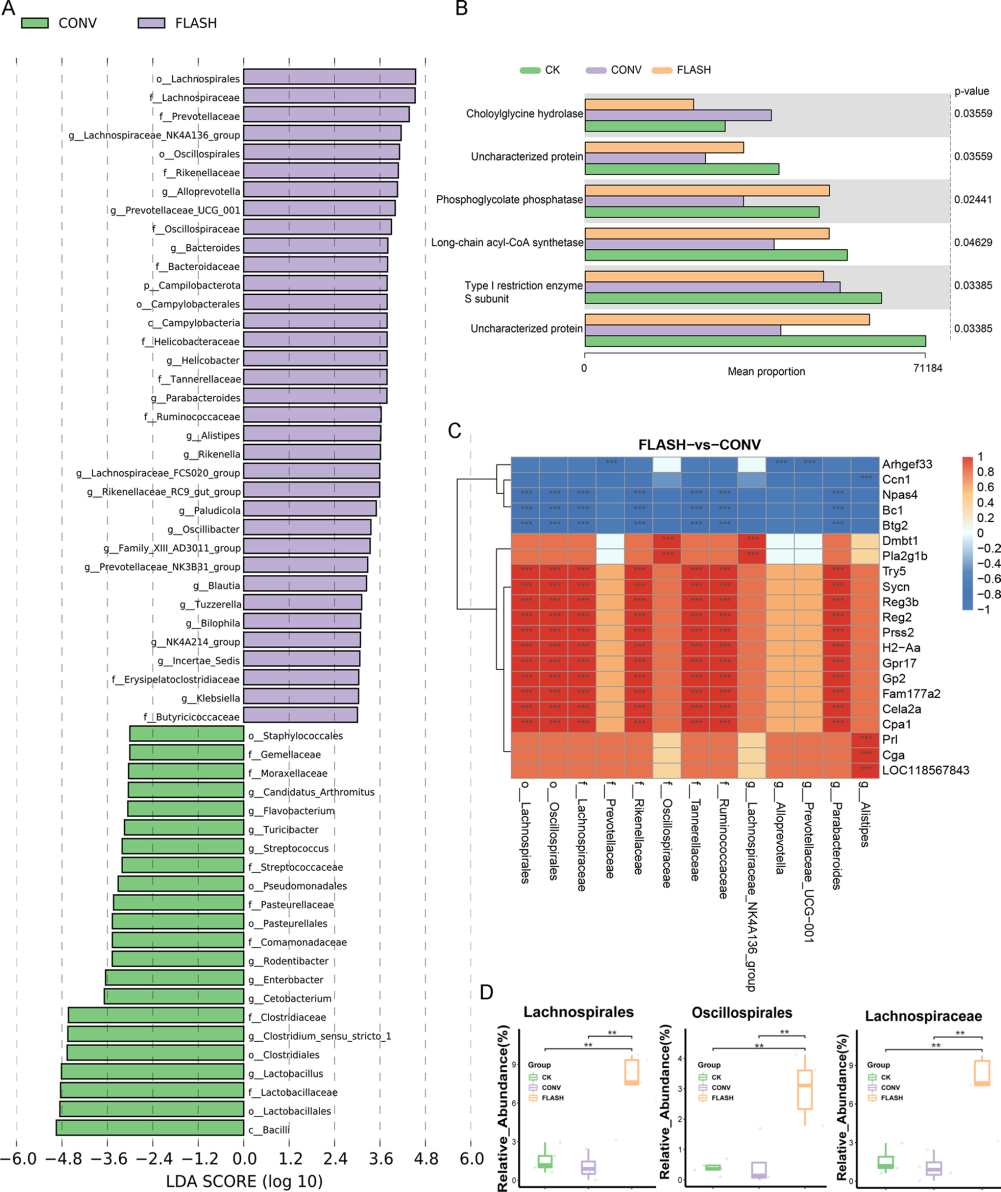
Analysis of microbial flora changes and correlation with transcriptome. **A** LDA score difference between CONV and FLASH irradiation groups; **B** Microbiome KEGG enrichment analysis, p_value from Kruskal–Wallis; **C** Correlation heatmap between differential microorganisms and transcriptome, Spearman correlation algorithm; **D** Abundance of differential flora in CK, CONV, and FLASH groups. “*” means p < 0.05, “**” means p < 0.01, “***” means p < 0.001)

The phylogenetic classification revealed the 15 most abundant phylum in the intestinal flora, with *Bacteroidetes* and *Firmicutes* being the two most abundant phylum across all analysis samples (Figure S3D). LEfSe analysis indicated that the *o_Lachnospirales* contributed the most to the differences in FLASH group (Fig. 5A). We listed the differential abundances of the three flora in different groups (Fig. 5D), then found that the abundance of probiotics in CONV group had a downward trend compared with that in CK group, but there was no statistical difference. However, in FLASH group significantly increased the abundance of probiotics compared with CK and CONV groups. Based on the KEGG function prediction, FLASH irradiation up-regulated some metabolism-related pathways, such as the phosphoglycolate phosphatase and long-chain acyl-CoA synthetase-related pathway, which is considered to be an important target of fatty acid metabolism, compared with CONV (p < 0.05) (Fig. 5B). Combined analysis of microbial diversity and transcriptomics revealed that taxa with increased abundance after eFLASH irradiation, such as *o_Lachnospirales, o_Oscillospirales, f_Lachnospiraceae, f_Rikenellaceae, f_Tannerellaceae, f_Ruminococcaceae,* were significantly positively correlated with Try5, Sycn, Reg3b, Reg2, Prss2, H2-Aa, Gpr17 and etc., while significantly negatively correlated with Npas4, Bc1and Btg2 (Fig. 5C).

## Discussion

Whole brain radiotherapy is a crucial method for the treating multiple brain metastases, but conventional irradiation can cause irreversible neurotoxicity. FLASH radiotherapy, known for reducing radiation toxicity of normal tissues, was also confirmed to be effective in our study, aligning with previous research findings (Simmons et al. 2019; Favaudon et al. 2014). In this study, we demonstrated that FLASH not only mitigates radiation-induced acute brain injury inflammation, but also provides long-term protective effects on cognitive and learning and memory abilities through histopathological analysis and behavioral evaluation. Interestingly, regarding motor function, we observed that CONV irradiation significantly reduced the movement capacity of mice at 1st week post-irradiation. However, no significant differences in movement capacity were noted among CK, CONV and FLASH groups at 3rd and 10th weeks post-irradiation. This suggests that the motor impairment of mice induced by CONV irradiation is recoverable over time. These findings are consistent with a study by Iturri et al. (2023), which used OF test to measure the motor and exploration ability of rats three months after intracranial radiation. Overall, our results validate the hypothesis that FLASH radiotherapy reduces radiation-induced acute brain injury and provides long-term cognitive protection while indicating that motor function impairment from CONV irradiation is recoverable.

Based on above results, we conducted mRNA sequencing on the whole brain samples of mice 1st week after radiotherapy to delve deeper into the molecular mechanism underlying the FLASH effect. KEGG enrichment analysis was performed after differential gene expression analysis in the CONV group and FLASH group. Notably, compared with CONV, the up-regulated genes in FLASH group were predominantly enriched in the digestive system and various metabolic pathways. In recent years, increasing studies have explored the intricated relationship between the brain and the gastrointestinal system. Numerous central nervous system diseases, such as Parkinson's disease and Alzheimer's disease, have been found to be closely related to the digestive system (Bove and Travagli 2019; Agirman et al. 2021; Ye et al. 2021). While research on the “brain-gut axis” remains largely inconclusive, it offers novel insights. The up-regulation of digestive system-related gene following FLASH irradiation suggests a potential enhancement in the interaction between the nervous system and the digestive system. In this intricate process, gut microbiota plays an pivotal role (Hu et al. 2023). Therefore, differences in the radiation response of intestinal microorganisms under conventional dose irradiation may offer evidence supporting the effectiveness of FLASH irradiation.

The gut microbiota play a vital role in the gut-brain axis, contributing significantly to emotional processing, behavioral regulation, metabolic processing and energy production (Nogacka et al. 2019; Gilbert et al. 2018). However, gut microbes are susceptible to environmental influences like radiation exposure (Kim et al. 2015; Guo et al. 2020). Wang et al. (2024) demonstrated that X-ray intracranial irradiation can affect the community structure of intestinal microorganisms. In our study, we used the ultra-high dose rate and conventional dose rate electron beams for a single 10 Gy whole-brain irradiation to investigate the differential effects of CONV and FLASH irradiation on the intestinal flora in mice at the later stage. Subsequently, we performed 16S rRNA sequencing analysis on the intestinal contents of mice at the 10th week post-irradiation. Our findings suggest that FLASH irradiation has minimal effect on the structure of the gut microbiota, while increasing the abundance of various “probiotics”, such as *Lachnospiraceae*, *Ruminococcaceae*, *Oscillospiraceae* and so on. Studies have reported that the gut microbiota can affect the activation of glial cells through metabolic signaling pathways such as lipopolysaccharide (LPS) and fatty acids (Rothhammer et al. 2018). In 2020, an article published in Science unveiled that *Lachnospiraceae* can mitigate radiation toxicity by producing SCFAs through whole-body irradiation (Guo et al. 2020), exerting radiation protection effects. Moreover, its genus was found to be significantly negatively correlated with the severity of depressive symptoms (Ye et al. 2021). Similarly, *Oscillospiraceae,* which has been linked to alleviating inflammatory diseases, is considered a potential next-generation probiotic for addressing metabolic syndrome, also produces SCFAs (Konikoff and Gophna 2016). SCFAs have the ability to traverse the blood–brain barrier (BBB), facilitating their transfer from the colon, where they are produced, to the brain. Through functional prediction of the differential flora, the our results indicated that FLASH primarily up-regulated microbial metabolic-related pathways. Our joint transcriptomics analysis revealed a significantly positive correlation between these “probiotics” and genes like Reg2 and Reg3b, known for their involvement in neuronal regeneration (Tebar et al. 2008), and a significant negative correlation with genes like Npas4, implicated in inhibitory synapses redistribution (Bloodgood et al. 2013).

Therefore, we conclude that the augmented abundance of “probiotics” such as *Lachnospiraceae* induced by eFLASH irradiation might contribute to the attenuation of radiation-induced neurotoxicity. Consequently, the alteration in intestinal microorganisms presents a novel hypothesis for understanding the mechanism behind the FLASH effect. Ongoing research endeavors seek to delve deeper into this area.

However, there are still certain limitations to address. Our study solely integrates behavioral, transcriptomic, and microbiome analyses to elucidate the divergence in radiation-induced effects on intestinal flora between conventional and eFLASH irradiation, without further substantiating the specific pathways of the gut-brain axis involved in reducing radiation toxicity. Therefore, forthcoming investigations will delve into unraveling the precise mechanism of intestinal microorganisms contributing to the FLASH effect.

## Conclusions

**Fig. 6:**
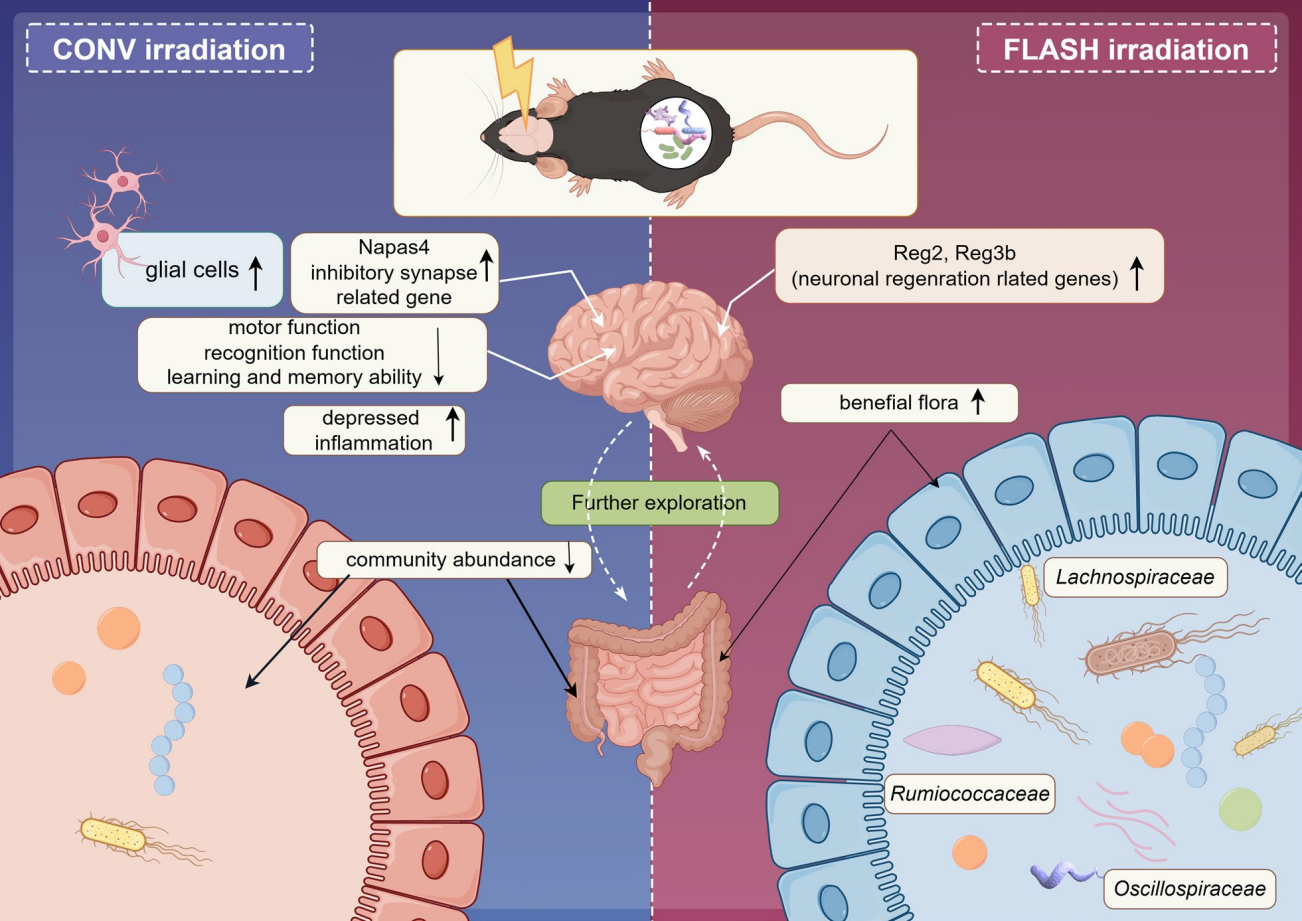
Graphical abstract (by Figdraw 2.0)

Our study represents the first investigation into the effects of whole-brain eFLASH irradiation on the distal intestinal microbiota in mice (Fig. 6). Through a comprehensive analysis of behavioral and omics data, eFLASH irradiation was found to mitigate acute radiation injury in mice and confer long-term protective effects on cognitive function and spatial learning and memory. Moreover, eFLASH irradiation up-regulated genes related to the digestive system and increased the abundance of intestinal “probiotics”. KEGG function prediction and correlation analysis revealed the involvement of these “probiotics” in fatty acid metabolism related pathways, along with significant positive correlations with the Reg family (associated with the development and regeneration of motor neuron subsets) and significant negative correlations with Npas4 (an inhibitory synapse-related gene). These findings represent an initial step in experimental research on the role of the eFLASH effect in the gut-brain axis and offer valuable insights for further exploring the mechanisms underlying FLASH irradiation.

## Supplementary Information

Supplementary Material 1: Figure S1. HE staining and OF test (A. HE staining 3rd days after irradiation; B. OF test in the 3rd week; C. OF test in the 10th week; D. The rotarod test in the 10th week.

Supplementary Material 2: Figure S2. GO and KEGG functional enrichment analysis.

Supplementary Material 3: Figure S3. Microbial community analysis (A-B. the sparse curve of the analyzed samples; C. The Rank-Abundance curve; D. The relative abundance of phylum.

Supplementary Material 4: Figure S4. Gene function analysis in radiation group compared with non-irradiated group (A. CONV vs CK; B. FLASH vs CK; C. GSEA enrichment analysis.

## Data Availability

No datasets were generated or analysed during the current study.

## Abbreviations

WBRT: Whole-brain radiotherapy
FLASH: Ultra-high dose rate
eFLASH: Electron-beam FLASH
COVN: Conventional radiotherapy
FLASH-RT: Flash radiotherapy
SCFAs: Short-chain fatty acids
OF: Open field test
NOR: Novel object recognition
RR: Recognition ratio
FST: Forced Swimming test
MWM: Morris water maze
PNT: Place navigation test
SPT: Spatial probe test
DEGs: Differential expression genes
KEGG: Kyoto Encyclopedia of Genes and Genomes
ASVs: Amplicon sequence variants
PCoA: Principal co-ordinates analysis
LDA: Linear discriminant analysis
LEfSe: LDA coupled with effect size measurements
