# Supplementary figures and images for: Electron-beam FLASH whole brain irradiation induced a unique changes of intestinal flora
Source: Mol Med. 2025 May 2;31:165. doi: 10.1186/s10020-024-01053-w (PMC12049017; doi:10.1186/s10020-024-01053-w)

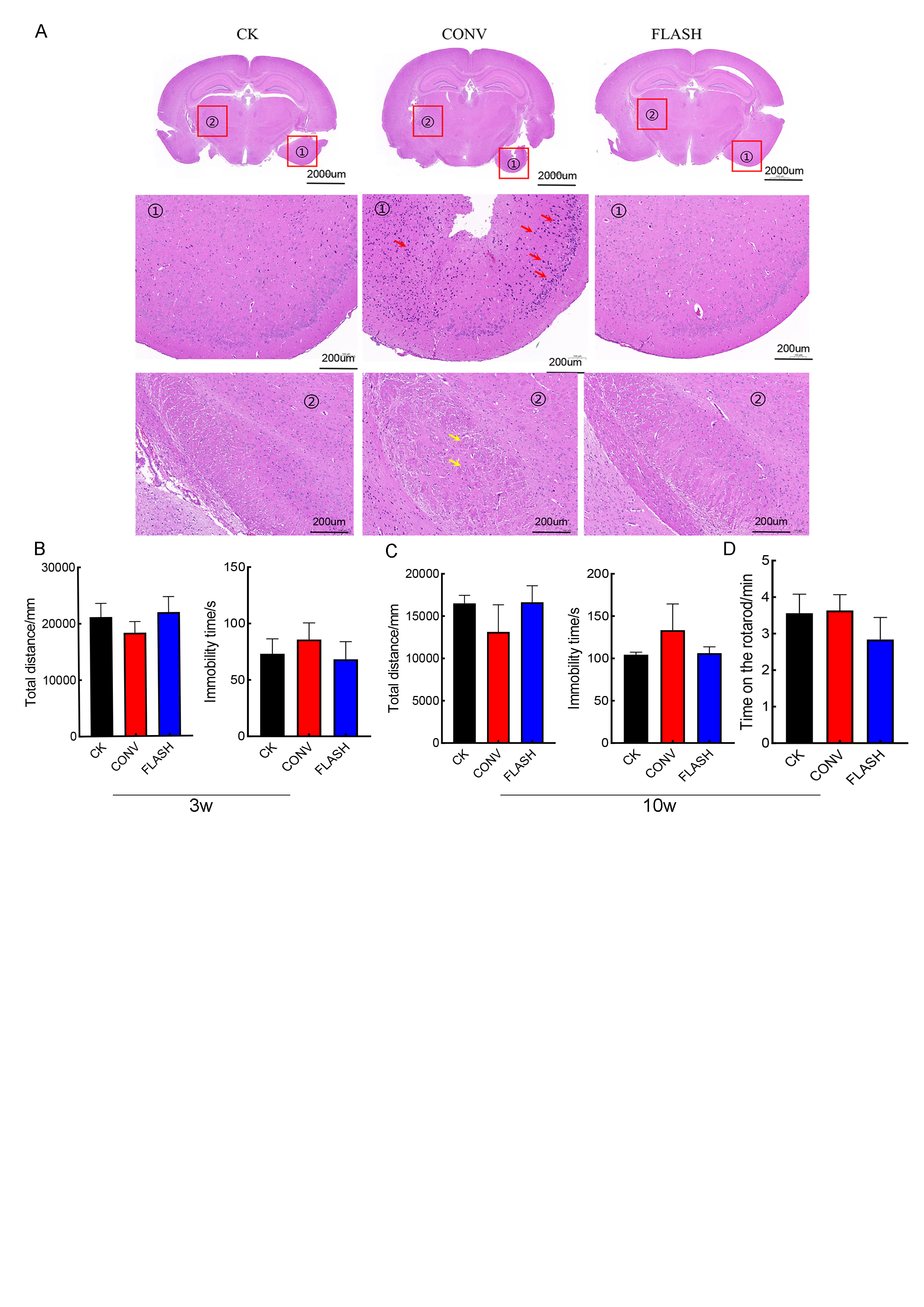

Supplement: Supplementary file 1 — Supplementary Material 1: Figure S1. HE staining and OF test (A. HE staining 3rd days after irradiation; B. OF test in the 3rd week; C. OF test in the 10th week; D. The rotarod test in the 10th week. [file 10020_2024_1053_MOESM1_ESM.tif]

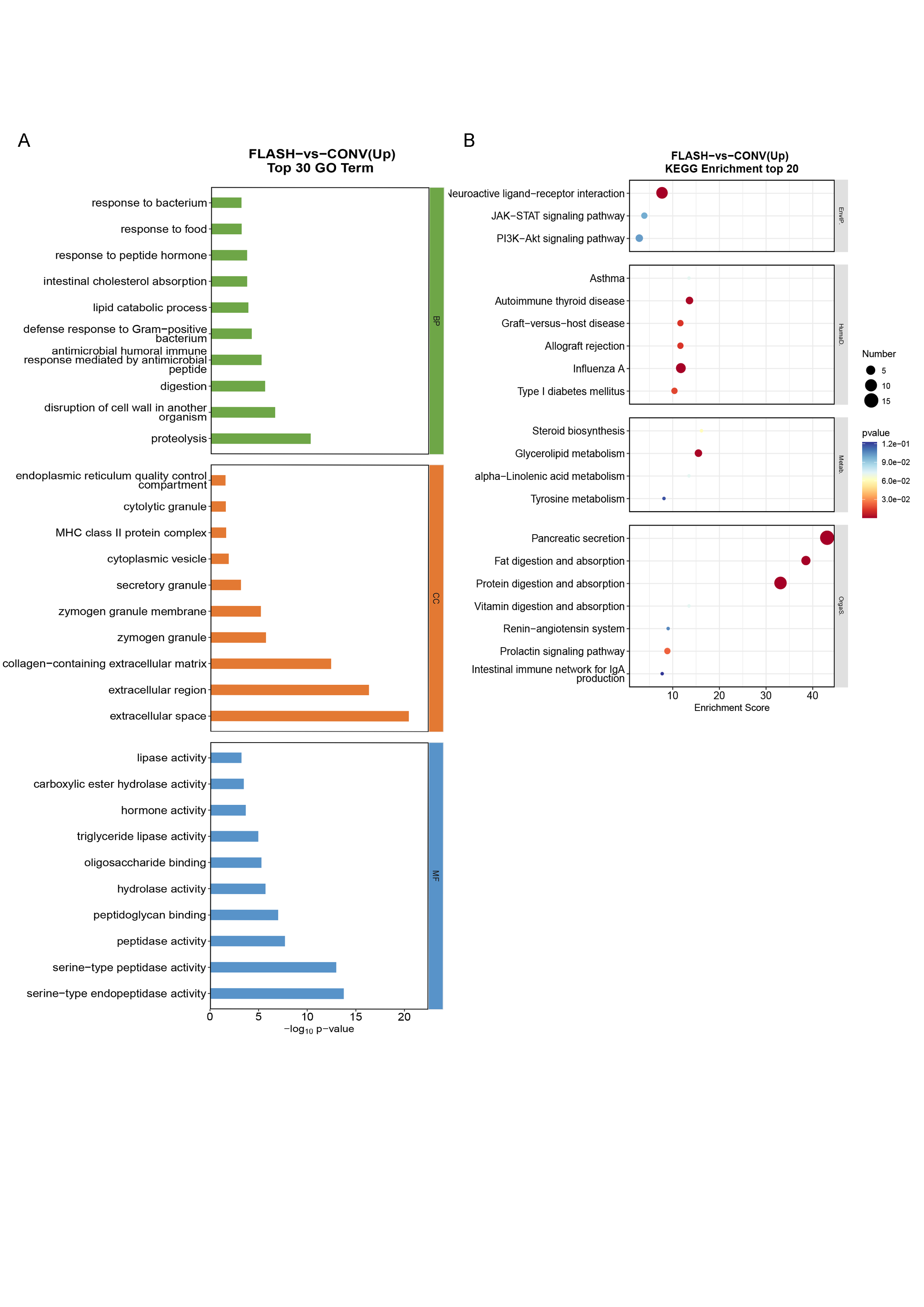

Supplement: Supplementary file 2 — Supplementary Material 2: Figure S2. GO and KEGG functional enrichment analysis. [file 10020_2024_1053_MOESM2_ESM.tif]

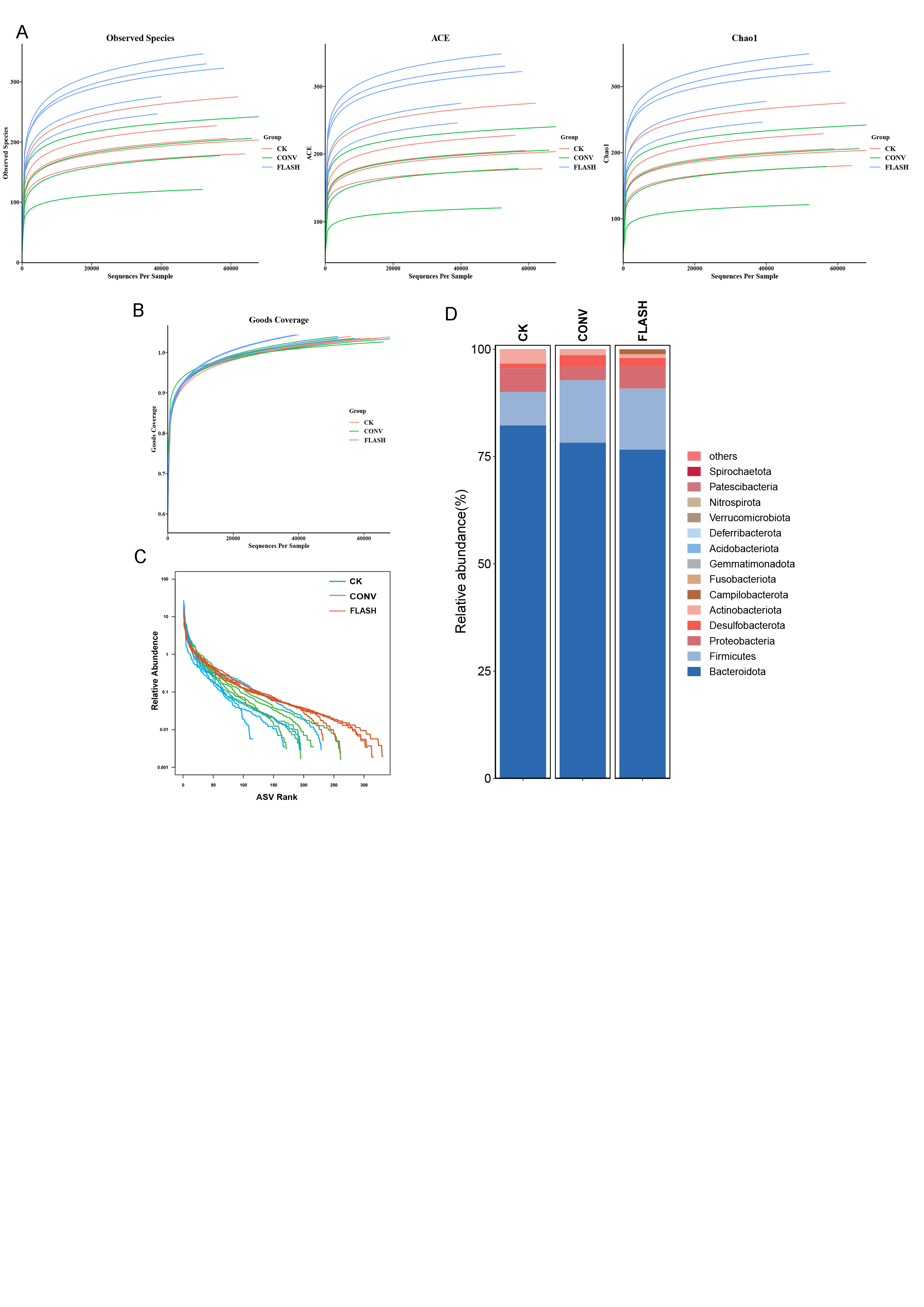

Supplement: Supplementary file 3 — Supplementary Material 3: Figure S3. Microbial community analysis (A-B. the sparse curve of the analyzed samples; C. The Rank-Abundance curve; D. The relative abundance of phylum. [file 10020_2024_1053_MOESM3_ESM.tif]

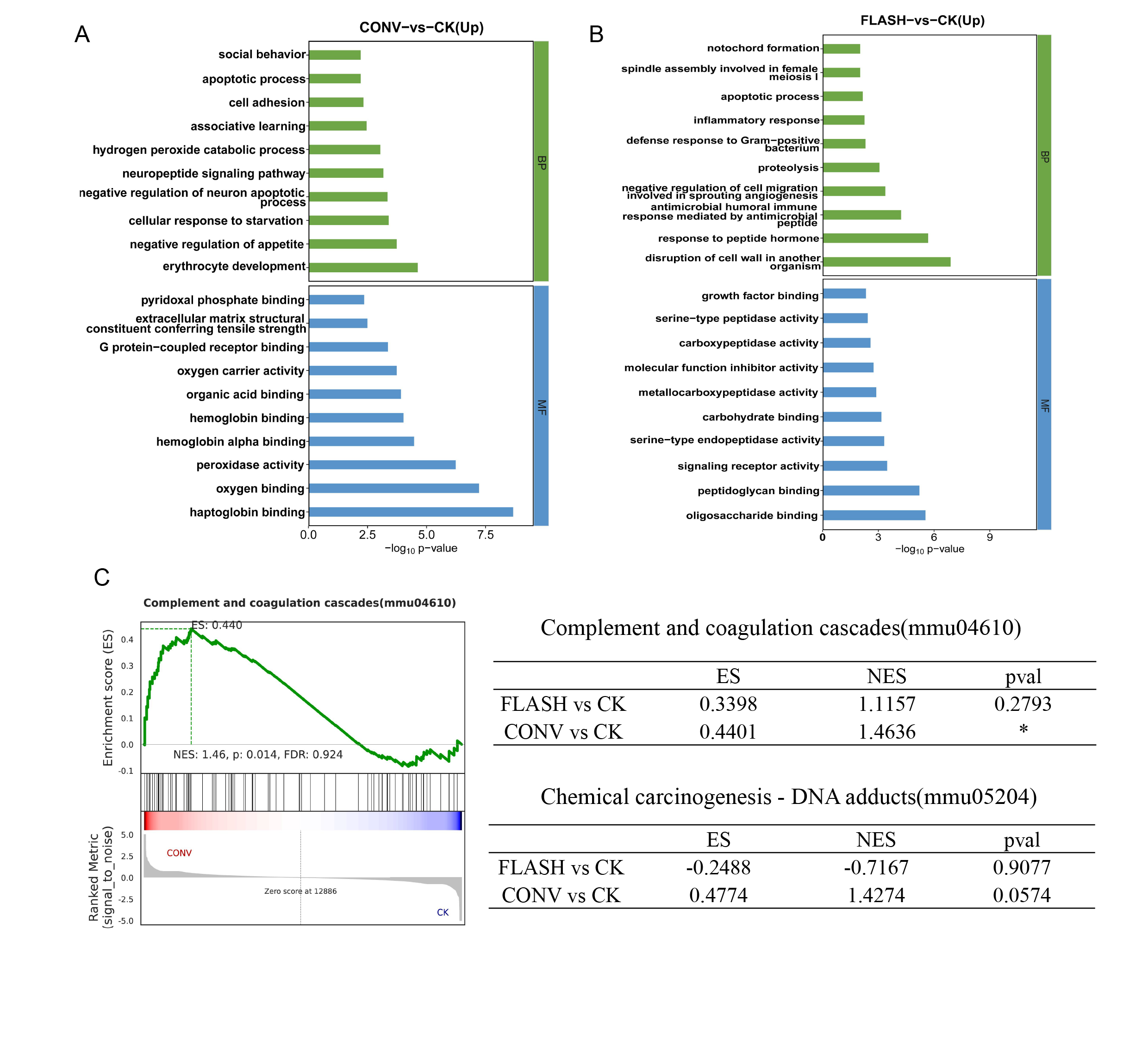

Supplement: Supplementary file 4 — Supplementary Material 4: Figure S4. Gene function analysis in radiation group compared with non-irradiated group (A. CONV vs CK; B. FLASH vs CK; C. GSEA enrichment analysis. [file 10020_2024_1053_MOESM4_ESM.tif]
